# Greater ecophysiological stress tolerance in the core environment than in extreme environments of wild chickpea (*Cicer reticulatum*)

**DOI:** 10.1038/s41598-024-56457-9

**Published:** 2024-03-08

**Authors:** Christopher P. Krieg, Duncan D. Smith, Mark A. Adams, Jens Berger, Niloofar Layegh Nikravesh, Eric J. von Wettberg

**Affiliations:** 1 University of Wisconsin, Madison, WI USA; 2 Swinburne University of Technology, Hawthorn, VIC Australia; 3 CSIRO, Agriculture and Food, Perth, WA Australia; 4 Department of Plant and Soil Science, University of Vermont, Burlington, VT USA

**Keywords:** Plant physiology, Abiotic

## Abstract

Global climate change and land use change underlie a need to develop new crop breeding strategies, and crop wild relatives (CWR) have become an important potential source of new genetic material to improve breeding efforts. Many recent approaches assume adaptive trait variation increases towards the relative environmental extremes of a species range, potentially missing valuable trait variation in more moderate or typical climates. Here, we leveraged distinct genotypes of wild chickpea (*Cicer reticulatum*) that differ in their relative climates from moderate to more extreme and perform targeted assessments of drought and heat tolerance. We found significance variation in ecophysiological function and stress tolerance between genotypes but contrary to expectations and current paradigms, it was individuals from more moderate climates that exhibited greater capacity for stress tolerance than individuals from warmer and drier climates. These results indicate that wild germplasm collection efforts to identify adaptive variation should include the full range of environmental conditions and habitats instead of only environmental extremes, and that doing so may significantly enhance the success of breeding programs broadly.

## Introduction

Global climate change is threatening food security for billions of people more than ever before in modern human history, particularly in developing regions^1–3^. Changes in climate are leading to increased temperatures and more frequent droughts, severely negatively impacting food production and yields^4,5^. For example, drought and heat waves in the last several years have significantly reduced the annual yield of major crops^6,7^ and the United Nations Food and Agriculture Organization (FAO) has declared climate change induced changes in crop abiotic conditions as one of the most urgent issues to modern agricultural practices (e.g.,^8^). Due to the reduced genetic variation induced by domestication^9,10^, many crop cultivars have been concurrently selected for increased yield and decreased resilience to biotic and abiotic stressors^11–13^. To combat the increase in stress vulnerability driven by reduced genetic variation, intense efforts have been paid to understanding the genetic variation in crop wild relatives and their utility to introgress new wild genetic material into established cultivars in the face of increasingly harsh climates^14–17^; as opposed to genetic engineering approaches (e.g.^13^). A common assumption behind the focus on identifying genetic resources in the wild relatives of crop cultivars is that it may come with adaptive ecophysiological variation that can be bred into cultivars to maintain or increase yield in stressful conditions relative to more vulnerable crop cultivars. The few studies that exist on ecophysiological variation in wild relatives of crops have demonstrated the power of ecophysiological tools to inform breeding programs^18–22^. For example, introgression of wild alleles has improved the drought tolerance of rice^23^, wheat^24^, and tomatoes^25^. Despite the power of physiological and genetic approaches to assess physiological variation that may be driven by genetic variation when used in tandem, many globally important crop wild relatives are still poorly characterized and the extant ecophysiological variation is unknown.

Crop legumes are globally important food crops and are especially important in rural and developing regions because they can be grown in relatively nutrient poor soils and can be rotated with other crops to increase soil nitrogen^26–28^. In many diets they are also critical sources of dietary protein, vitamins, minerals, and fiber. Chickpea is the third leading grain legume in global production, being particularly important in semi-arid tropical regions. Originally domesticated in the Fertile Crescent, chickpea spread to South and Central Asia, the Western Mediterranean, and the East African Highlands over the past few thousand years^29^. South Asia accounts for 75% of world chickpea production, and ~ 80% of its consumption, with India as the world’s leading producer, consumer, and importer of chickpea^30^. During the green revolution, increases in wheat yields pushed chickpea production southward, from Uttar Pradesh towards Andra Pradesh^31^. This has meant that chickpea production in India now occurs in drier and hotter regions than in the past. Similar processes have led to chickpea often being produced on marginal land in other regions, from Pakistan to Ethiopia^30^. Both current trends in climate changes and historical agricultural processes have pushed the production of chickpea into warmer and drier areas, exacerbating the need to understand extant physiological variation in wild relatives of cultivated chickpea.

The ultimate goal of many breeding programs is to produce cultivars with better ecophysiological stress tolerance^18,32,33^. However, most efforts to assess and preserve the extant diversity of crop wild relatives do not explicitly consider spatial physiological diversity and have instead focused solely on identifying and characterizing genetic diversity. For example, several approaches have been used to assess the association between genetic variation and climate variation, including LEA^34^, BayEnv^35^, Bedassle^36^, and Gradient Forests^37,38^. These approaches are leveraged in breeding approaches like Focused Identification of Germplasm Strategy (FIGS)^39,40^ that assume physiological traits are linked to the climate of origin. If this assumption holds^41^, these approaches can effectively target accessions from extreme environments for crosses and germplasm conservation strategies like Gap Analysis^39,40,42^. These genetics-focused approaches show the importance of understanding spatial genetic variation to make the identification of adaptive physiological variation more likely and more efficient^43–45^. However, the focus on targeting populations in extreme environments may miss useful adaptive trait variation in more moderate climates. Relatively few studies have characterized ecophysiological stress tolerances in crop wild relatives, which limits our understanding of where to find ecophysiological variation on the landscape for breeding efforts. More research is needed to determine where meaningful ecophysiological variation occurs across spatial and environmental scales.

**Figure 1:**
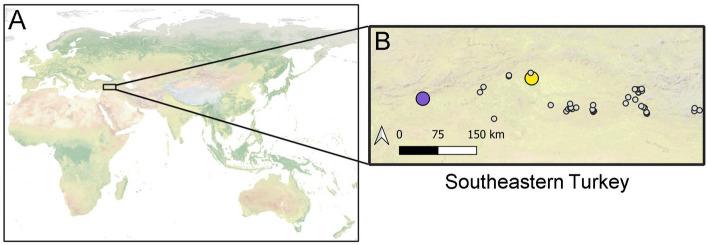
Geographic map of 47 wild populations of *Cicer reticulatum* across their native distribution in Turkey. Kalka (Kalka_070; Kalkan, Diyarbakir Province) is shown in yellow and Oyali (Oyali_107; Oyali, Adiyaman Province) is shown in purple. Grey points area background populations used to characterize the native environmental conditions of wild *Cicer reticulatum.*

**Figure 2:**
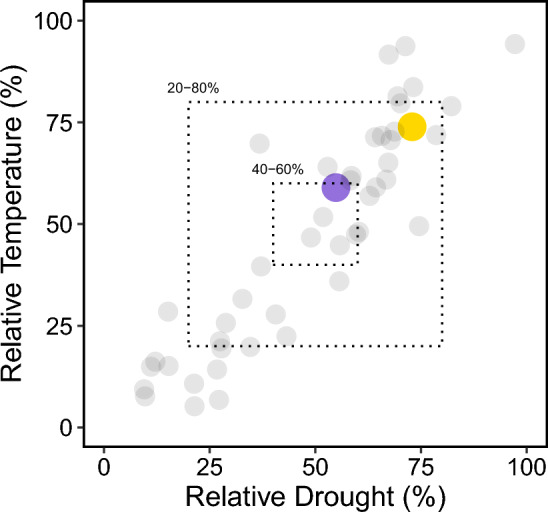
The mean percentile across temperature related variables (y-axis) and drought related variables (x-axis) for 47 wild populations of *Cicer reticulatum*. The moderate climate genotype, Oyali, is in purple and the more extreme climate genotype, Kalka, is in yellow. The inner dotted box indicates the core climate between the 40th and 60th percentiles and the outer dotted box indicates the more extreme percentiles between 20 and 80 percent. Kalka has a mean relative temperature percentile of 73.92 (± 2.01 s.e.) and a mean relative drought percentile of 72.9 (± 1.40 s.e.). Oyali has a mean relative temperature percentile of 58.97 (± 2.4 s.e.) and a mean relative drought percentile of 54.87 (± 3.02 s.e.). Raw percentiles across all variables for each site can be found in Supplementary File 1.

Increasingly harsh abiotic conditions are among the leading challenges for chickpea production globally^46,47^ and characterizing the physiological variation in abiotic stress responses is among the most important goals to improving the resilience of major crops like chickpea to climate change^46,48,49^. In particular, studies have shown that drought and heat stress are the two primary abiotic factors that most strongly impact the growth, phenology, and yield of chickpea cultivars and wild relatives^46,47,50–53^. Thus, the goal of many breeding programs is to capture adaptive trait variation and tolerance to drought and heat stress in genotypes of crop wild relatives that have the potential to be new resources for breeding climate resilient cultivars. Here, we test whether relatively extreme climates correspond to adaptive ecophysiological function and stress tolerance compared to individuals from more moderate climates. Specifically, we used a common garden and intensive assessments of ecophysiological responses to water and temperature in wild collections of *Cicer reticulatum* with distinct genetic backgrounds from the core and relative extreme parts of its natural range (Figs. 1, 2). We hypothesized that distinct genotypes would show differences in their response to water and temperature stress and that individuals from more extreme climates will show greater stress tolerance consistent with prevailing paradigms.

## Results

### Genotype comparison of leaf traits

**Figure 3:**
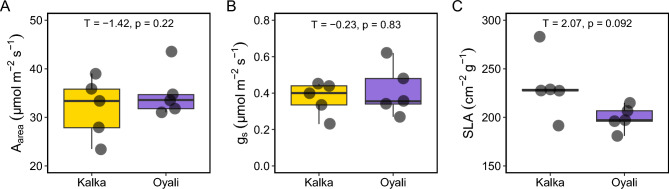
Trait comparisons between the more extreme Kalka (yellow) and more moderate Oyali (purple) genotypes; (**A**) Photosynthetic rate per area (A_area_), (**B**) stomatal conductance (g_s_), (**C**) specific leaf area (SLA). Group comparison statistics (e.g. T-statistic and p-value) are at the top center of each panel and asterisks indicate significant differences between genotypes. None were significant.

We did not detect any significant differences between the Kalka (genotype from a relatively extreme environment) and Oyali (genotype from a common environment) genotypes in photosynthetic rate per area (A_area_; T = -1.42, p = 0.22; Fig. 3A), stomatal conductance (g_s_; T = -0.23, p = 0.83; Fig. 3B), or specific leaf area (SLA; T = 2.07, p = 0.092; Fig. 3C) under non-experimental conditions (see “Methods”).

### Pressure–volume curves

**Figure 4:**
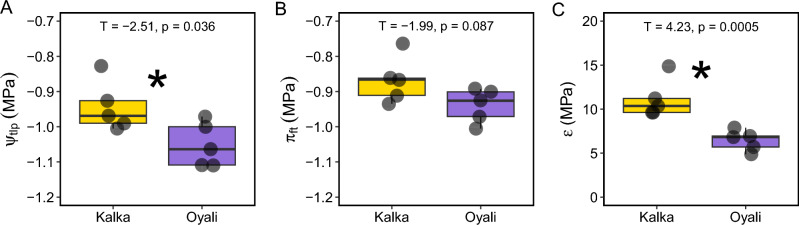
Comparison of pressure–volume curve parameters between the more extreme Kalka (yellow) and more moderate Oyali (purple) genotypes; (**A**) turgor-loss point (Ψ_tlp_), (**B**) osmotic potential at full turgor (π_ft_), (**C**) bulk elastic modulus (ε). Group comparison statistics (e.g., T statistic and p-value) are at the top center of each panel and asterisks indicate significant differences between genotypes.

There were significant differences in pressure–volume curve parameters between the (more extreme) Kalka and (more moderate) Oyali genotypes. The mean turgor-loss point was significantly lower in the Oyali than Kalka (T = -2.51. p = 0.036; Fig. 4A). We did not find any difference in the osmotic potential at full turgor between genotypes (T = -1.99. p = 0.087; Fig. 4B). The bulk elastic modulus was significantly higher in the Kalka genotype compared to the Oyali genotype (T = 4.23, p = 0.005; Fig. 4C).

### ***Temperature*** × ***A/C***_***i***_*** curves***

**Figure 5:**
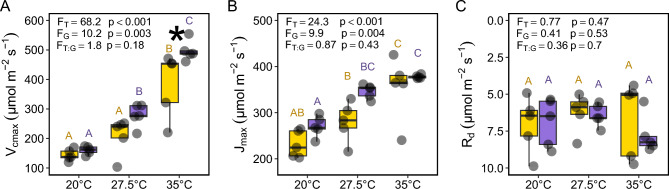
Comparison of A/C_i_ curve parameters between the more extreme Kalka (yellow) and more moderate Oyali (purple) genotypes across three temperature treatments, 20 °C, 27.5 °C, and 35 °C; (**A**) maximum carboxylation rate (V_cmax_), (**B**) maximum electron transport rate (J_max_), (**C**) estimated dark respiration rate (R_d_). Capital letters indicate statistical groupings within a genotype and across temperature treatments. Asterisks indicate significant differences between genotypes within a temperature treatment. F-ratios are reported for factors temperature (F_T_), genotype (F_G_), and their interaction (F_T:G_) followed by their p-values in the top left corner of each panel.

Two-way ANOVAs detected significant effects of temperature and genotype on photosynthetic responses to three temperature treatments (Fig. 5A–C). The effect of temperature on maximum rates of carboxylation (V_cmax_) was significant and positively associated with increasing temperature (F_T_ = 68.2, p < 0.001; Fig. 5A). Similarly, both genotypes significantly increased V_cmax_ with increasing temperatures (F_G_ = 10.2, p = 0.003; Fig. 5A). The model did not detect any significant interaction between temperature and genotype (F_T:G_ = 1.8, p = 0.18; Fig. 5A). However, pairwise comparisons indicated differences between genotypes at and 35 °C (Fig. 5A).

The effect of temperature on maximum rates of electron transport (J_max_) was significant and positively associated with increasing temperature (F_T_ = 24.3, p < 0.001; Fig. 5B). Similarly, both genotypes significantly increased J_max_ with increasing temperatures (F_G_ = 9.9, p = 0.004; Fig. 5B). The model did not detect any significant interaction between temperature and genotype (F_T:G_ = 0.87, p = 0.43; Fig. 5B).

We did not find any effect of temperature (F_T_ = 0.77, p = 0.47; Fig. 5C), genotype (F_G_ = 0.41, p = 0.53; Fig. 5C), or their interaction (F_T:G_ = 0.41, p = 0.7; Fig. 5C) on estimated dark respiration rates (R_d_).

## Discussion

The latest breeding approaches target populations of crop wild relatives in marginal or relatively extreme environments as a strategy to increase the probability of capturing wild adaptive genetic variation that can be introgressed into stress intolerant cultivars^39,40,42^. However, such an approach may come with some caveats. For example, the approach of sampling from the extremes may miss valuable physiological variation at more moderate climates and in areas more suitable for most individuals of the wild relative. We took a conservative approach to testing this assumption by choosing a genotype near the relative environmental extreme (Kalka) and a genotype in environmental conditions that are more typical of *Cicer reticulatum* (Oyali) (see “Methods”; Figs. 1, 2). Because drought and heat are the most important factors negatively impacting chickpea production and productivity in agricultural systems globally, we characterized the responses of two distinct genotypes to water and temperature stress.

We found that the two wild genotypes significantly differed in their responses to water-stress. In particular, the more moderate Oyali genotype exhibited a significantly lower turgor loss point (Ψ_tlp_) indicating that there is genetic variation for drought stress tolerance (Fig. 3A). Our analyses also show that the increased drought tolerance exhibited by the Oyali genotype may be driven by an increase in the elastic properties of cell wall membranes, as indicated by the bulk elastic modulus (ε), rather than the osmotic potential at full turgor (π_ft_) (Fig. 3B,C). Our data suggest there may be genetic variation underlying the elastic properties of cell walls in wild chickpea and that this trait may be a main driver of cell responses to declining water potential in leaves of *Cicer reticulatum*. This may be particularly interesting to breeders if breeding for structural traits, like the properties of cell walls, is a more efficient target of breeding efforts (as a proxy for physiological responses) compared to targeting complex molecular and biochemical processes directly. Several additional micro-anatomical characteristics like cell wall thickness, cell density, and palisade arrangement have been shown to be strongly correlated with macro level leaf traits like specific leaf area (SLA) in some systems^54,55^. Specific leaf area has been used as a broad indicator of ecological strategy, particularly in terms of growth and allocation^56–58^. Variation in SLA can also impact the diffusion of gases into and out of leaves including strong impacts on water-use by impacting the resistance of liquid and vapour fluxes, when low SLA is associated with thick and/or dense leaves^54,59–63^. However, we found no difference between SLA measured among wild genotypes in our common garden (Fig. 3), suggesting that SLA may not be a good predictor or indicator of leaf-level physiological function in wild chickpea.

Drought and heat stress are strongly correlated across broad spatial scales (e.g., Fig. 1) and the intensity of each are predicted to increase into the future^64–66^. Our analyses of photosynthetic responses to temperature revealed that the two genotypes are both strongly impacted by increased temperatures in similar ways up to 35 °C. A/C_i_ curve parameters V_cmax_ and J_max_ increased with increasing temperature (Fig. 5A,B) suggesting that the enzymatic and chemical optimum for V_cmax_ and J_max_ in these genotypes is at least 35 °C and possibly higher. Our temperature maxima were not able to damage photosynthetic machinery enough to cause declines in V_cmax_ or J_max_, however, in the highest temperature treatment of 35 °C, Oyali and Kalka genotypes became distinct in their apparent V_cmax_. Specifically, the Oyali genotype from moderate native temperatures was able to achieve a higher V_cmax_ than the Kalka genotype with much higher native temperatures in the 35 °C treatment (Fig. 5A), suggesting the more moderate Oyali genotype may have a greater capacity for temperature acclimation. Our experiments detected shifts in V_cmax_ and J_max_, and the difference in capacity for near-term acclimation between genotypes in V_cmax_, however, the potential shifts in species distributions and physiological function into the future is a function of both acclimation and adaptation. Understanding the adaptive potential of crop wild relatives is a rapidly growing frontier in research at the nexus of basic and applied botany. Further research is needed to understand the adaptive capacity of wild chickpea and how the capacity of adaptive evolution and/or plasticity may differ between genotypes. Such a characterization of physiological capacities would be of extraordinarily high value to breeding efforts to introgress targeted physiological variation of wild relatives into crop cultivars.

Finding that the genotype from the much drier and warmer part of the species range (Kalka) exhibited lower tolerance to water stress, as well as less capacity for photosynthetic acclimation to temperature was opposite to our hypothesis and counter to the prevailing paradigm that individuals and genotypes from more extreme environments should exhibit greater adaptive physiology. Our results suggest that efforts to identify adaptive trait variation should focus on the entire range of a species and not only the environmental extremes. In addition, these data show that genetic approaches to identify adaptive trait variation in crop wild relatives must include ecophysiology or risk missing opportunities to discover key physiological traits important to breeding more climate resilient crop cultivars. However, it is important to acknowledge the limitations of our study due to the comparison of two genotypes. For example, it is not possible to disentangle in strength of influence of climate on physiological function and partition the variance in physiological traits among sites and between sites. Future studies could include a broader sampling of accessions to better characterize the role of native climate and soil type in determining variation in physiological phenotypes. Moreover, the link between our discovery of differential stress tolerance and breeding targets such as biomass, yield, harvest index, and seed filling capacity are unclear. Making direct linkages between ecophysiological research and agronomic traits will be critical to designing more effecting strategies for identifying adaptive physiological traits and favourable agronomic traits across the native range of wild crop relatives.

## Methods and materials

### Common-garden

Our wild accessions were selected from those in von Wettberg et al.^22^. Specifically, JB, CPK, DDS, and MAA selected two genotypes that originated from sites with different climatic conditions, one from Kalkan (Kalkan, Diyarbakir province; accession name Kalka_70) that represents some of the drier climatic conditions where chickpea is found in this region, while the other site, Oyali (Besni, Adiyaman Province; accession name Oyali_107) represents a more typical climatic site within the natural range of *Cicer reticulatum* (Figs. 1, 2). JB grew seeds from these accessions in a common garden set-up where seeds of each accession were planted in 11-L pots containing 8 L of a mixture of sandy loam and coconut coir. Plants were maintained in a water-cooled glass house (mean temperature = 20 °C) under ambient photoperiod (12–14 h) in Perth, Australia. Plants were watered three times a week by an automatic irrigation system. All pots were randomly arranged in a grid in the glasshouse. Seeds were planted in October and were at least 2 months old at the time of measurements.

Field-collection campaigns were conducted in accordance with provincial guidelines and regulations and with written permission by Turkish government. All plant specimens were inspected and identified to the species rank by Josie Piggin from the International Center for Agriculture Research in the Dry Areas (ICARDA). Vouchers are deposited at Akdeniz University.

### Characterizing climates

CPK characterized the macroclimatic niche of wild *Cicer reticulatum* using the geographic coordinates form wild populations in von Wettberg et al.^22^. Geographic occurrence records were thinned to ~ 1 km^2^ resulting in 34 unique sites across the range of *Cicer reticulatum*. Environmental data were extracted from global environmental rasters from Chelsa^67^, TerraClimate^68^, and SoilTemp^69^. See the “Supplementary Materials” for a complete list of environmental variables (Tables S1, S2). Geographic and climate occupancy were visualized using R (R Core Team, 2023) and QGIS (QGIS Development Team, 2023).

### Photosynthetic responses to temperature

To understand how temperature may differentially impact the efficiency of photosynthesis biochemistry of genotypes from contrasting climates, CPK, DDS, and MAA measured physiological responses to temperature and CO_2_ (i.e., A/C_i_ curves). Prior to measurement, plants were acclimated to 20 °C, 27.5 °C, or 35 °C with daytime PAR of 550 μmol m^-2^ s^-1^ inside a Conviron PGC Flex growth cabinet (Conviron Environments Ltd. Grovedale, Victoria, Australia). After at least 24 h acclimation, photosynthetic responses to changes in CO_2_ were measured using an LI-6400 infrared gas analyzer (Li-6400, Li-Cor Inc., NE, USA). Cuvette light conditions were set to 1300 μmol m^-2^ s^-1^ PAR. The block temperature was set to the growth cabinet temperature: 20 °C (mean T_leaf_ was 21.6 °C, sd = 0.42; mean cuvette VPD was 1.2 kPa, sd = 0.14); 27.5 °C (mean T_leaf_ was 29 °C, sd = 0.65; mean cuvette VPD was 2.2 kPa, sd = 0.20); or 35 °C (mean T_leaf_ was 35.8 °C, sd = 0.48; mean cuvette VPD was 3.8 kPa, sd = 0.35). We recorded net photosynthetic rate across a range of CO_2_ concentrations with at least seven setpoints between 50 and 2000 ppm (typically 400, 200, 100, 50, 400, 800, 1600, 2000).

To fit photosynthetic response curves to CO_2_ concentrations and estimate the maximum carboxylation rate (V_cmax_), the maximum electron transport rate (J_max_), and the dark respiration rate (R_d_), DDS and CPK used an optimization procedure as implemented by Lemoine^70–72^. Photosynthetic responses to CO_2_ concentrations were interpreted with the Farquhar-von Caemmerer-Berry model of carbon fixation in C3 plants^73,74^. See the “Supplementary Materials” for a complete list of statistical output (Table S3).

### Pressure–volume curves

To understand how water availability may differentially impact the survival of genotypes from contrasting climates, CPK and DDS conducted pressure–volume curves on the same two genotypes that were investigated for photosynthetic responses to temperature. Individual plants were grown in well-watered conditions and watered the night before pressure–volume curve measurements. We selected leaflets from 5 individuals of each genotype and placed them in individual Whirl–Pak bags to slow dehydration (Whirl–Pak, Nasco, Fort Atkinson, Wisconsin, USA). The fresh mass was recorded using a balance and initial water-potential using a Scholander pressure chamber (PMS Instrument Company,

Albany, OR, USA). Plants were repeatedly measured for their water-potential and plant mass while individuals were allowed to dehydrate on a benchtop. Whirl–Pak bags were loosened or removed to manipulate the rate of dehydration. Once leaflets had sufficiently passed their turgor-loss point (which was determined by visually inspecting the measurement data and typically occurred after about 10 measurement points), leaflets were placed in a drying oven at 70 °C for 36 h. Dried leaflets were weighed using a balance and their dry mass was recorded.

Final pressure–volume curve data and parameters were analyzed by CPK following Tyree and Hammel^75^. Here we focused on three main parameters: turgor-loss point (Ψ_tlp_), osmotic potential at full turgor (π_ft_), and bulk elastic modulus (ε). Briefly, the turgor-loss point (Ψ_tlp_) represents the cell water potential at which the cell pressure potential equals zero. Previous work has shown that the turgor-loss point is linked to stomatal closure and wilting during drought^76^, and broad ecological adaptation to water-availability^77–79^. The osmotic potential at full turgor (π_ft_), and bulk elastic modulus (ε) are two key properties of plant cells that impact plant cell turgor pressure and water potential. The osmotic pressure of cells is directly related to the ability of cells to absorb and release water through osmosis^80,81^. The bulk elastic modulus is a metric of the mechanical properties of cell walls and quantifies the relationship between a change in pressure potential for a given loss of water, where a lower bulk elastic modulus indicates a more elastic cell wall^82–84^.

### Specific leaf area

Images were taken of the fresh leaflets by CPK and DDS using a digital camera, before going into the drying oven (see above). Fresh leaflet area was calculated in ImageJ (Wayne Rasband/NIH, Bethesda, MD, USA). Samples were weighed after being dried (see above) and their dry mass was recorded. Specific leaf area (SLA) was calculated from the ratio of fresh area and dry mass (cm^2^/g). Several studies have shown that variation in SLA integrates multiple underlying axes of trait function^57^ and can be driven by broad ecological and environmental gradients^85–88^.

### Data analysis

All data analyses were performed in the R computing environment (R Core Team, 2022) by CPK, DDS, and/or NLN. The *dplyr* package was used for data manipulation and organization^89^. To test for the effect of genotype in our physiological trait measures (Figs. 2, 3), we performed a Student's t-test using the *rstatix* package^90^. To test the impact of temperature, genotype, and their interaction on A/C_i_ curve parameters (Fig. 4) we performed a two-way ANOVA and pairwise comparisons between genotypes and temperatures made using a Tukey’s HSD test with the *car* package^91^. Data were visualized with the *ggplot2* package^92^. All data used to create figures can be found in Supplementary File 1.

### Supplementary Information

Supplementary Information.

## Data Availability

The data supporting the results and code used to make figures are archived in Zendo (https://zenodo.org/records/10403071).
