# Supplementary material for: Greater ecophysiological stress tolerance in the core environment than in extreme environments of wild chickpea (Cicer reticulatum)
Source: Sci Rep. 2024 Mar 8;14:5744. doi: 10.1038/s41598-024-56457-9 (PMC10923935; doi:10.1038/s41598-024-56457-9)
Supplement: Supplementary file 1 — Supplementary Information. [file 41598_2024_56457_MOESM1_ESM.docx]

**Supplementary Information**

Greater ecophysiological stress tolerance in the core environment than in extreme environments of wild chickpea (*Cicer reticulatum*)

Christopher P. Krieg^1^*, Duncan D. Smith^1^, Mark A. Adams^2^, Jens Berger^3^,

Niloofar Layegh Nikravesh^4^, Eric J. von Wettberg^4^

^1^ University of Wisconsin, Madison, WI, USA

^2^ Swinburne University of Technology, Hawthorn, VIC, Australia

^3^ CSIRO, Agriculture and Food, Perth, WA, Australia

^4^ University of Vermont, Department of Plant and Soil Science, Burlington, VT, USA

*corresponding: ckrieg@wisc.edu

**Figures and Tables**:

**Table S1**. Full list of environmental variables and their sources.

**Table S2**. The percentile position of Kalka and Oyali in climate space relative to range-wide conditions for temperature and drought related variables.

**Table S3**. Full statistical output of Tukey pairwise comparisons from photosynthetic responses to temperature.

**File S1**. Data used in this study.

**Table S1**. An expanded table of 15 environmental used to characterize the temperature and water-availability within the natural range of Cicer reticulatum. To characterize the temperature conditions, we gathered 3 air temperature variables from CHELSA ^1^, and 3 soil temperature variables from SoilTemp ^2^. To characterize the water-availability as a proxy from drought conditions, we gathered 3 soil moisture variables from TerraClimate ^3^ and 6 variables related to atmospheric water-demand from CHELSA ^1^.

| **Short Code** | **Variable Name** | **Source** |
| --- | --- | --- |
| atemp.avg | mean annual temp | CHELSA |
| atemp.max | max temperature of warmest month | CHELSA |
| atemp.min | min temperature of coldest month | CHELSA |
| stemp.avg | mean annual soil temp | Lembrechts et al. |
| stemp.max | max soil temperature of warmest month | Lembrechts et al. |
| stemp.min | min soil temperature of coldest month | Lembrechts et al. |
| sm.avg | mean annual soil moisture | TerraClimate |
| sm.max | max monthly soil moisture | TerraClimate |
| sm.min | min monthly soil moisture | TerraClimate |
| vpd.avg | mean annual vapor pressure deficit | CHELSA |
| vpd.max | max monthly vapor pressure deficit | CHELSA |
| vpd.min | min monthly vapor pressure deficit | CHELSA |
| pet.avg | mean annual potential evapotranspiration | CHELSA |
| pet.max | max monthly potential evapotranspiration | CHELSA |
| pet.min | min monthly potential evapotranspiration | CHELSA |

**Table S2**. The ranking of Kalka and Oyali among 48 sites for all 16 variables that were used to calculate their mean rank across the 6 temperature (red) and 9 drought (blue) related variables (see Figure 1 in Main text).

| **Short Code** | **Oyali** | **Kalka** |
| --- | --- | --- |
| atemp.avg | 52.0% | 62.5% |
| atemp.max | 47.9% | 91.6% |
| atemp.min | 52.0% | 60.4% |
| stemp.avg | 56.2% | 81.2% |
| stemp.max | 52.0% | 64.5% |
| stemp.min | 93.7% | 83.3% |
| sm.avg | 98.0% | 58.8% |
| sm.max | 87.5% | 58.4% |
| sm.min | 98.0% | 58.4% |
| vpd.avg | 37.5% | 72.9% |
| vpd.max | 35.4% | 93.7% |
| vpd.min | 50.0% | 66.6% |
| pet.avg | 33.3% | 89.5% |
| pet.max | 8.3% | 66.6% |
| pet.min | 45.8% | 91.6% |

**Table S3**. The full output from Tukey all pairwise comparisons of three photosynthetic responses (V_cmax_, J_max_, and R_d_) for two genotypes (Kalka_070 in yellow and Oylai_107 in purple), across three temperature treatments (20°C, 27.5°C, and 35°C). Statistical significance is indicated by *p*-values in the right most column.

| **Trait** | **Temp (**°**C)** | **Genotype** | **Temp (**°**C)** | **Genotype** | ***p*-value** |
| --- | --- | --- | --- | --- | --- |
| V_cmax_ | 27.5 | Kalka_070 | 20 | Kalka_070 | 0.47 |
| V_cmax_ | 35 | Kalka_070 | 20 | Kalka_070 | **0.000008** |
| V_cmax_ | 20 | Oyali_107 | 20 | Kalka_070 | 0.99 |
| V_cmax_ | 27.5 | Oyali_107 | 20 | Kalka_070 | **0.011** |
| V_cmax_ | 35 | Oyali_107 | 20 | Kalka_070 | **0.000000** |
| V_cmax_ | 35 | Kalka_070 | 27.5 | Kalka_070 | **0.00067** |
| V_cmax_ | 20 | Oyali_107 | 27.5 | Kalka_070 | 0.74 |
| V_cmax_ | 27.5 | Oyali_107 | 27.5 | Kalka_070 | 0.40 |
| V_cmax_ | 35 | Oyali_107 | 27.5 | Kalka_070 | **4^E-07^** |
| V_cmax_ | 20 | Oyali_107 | 35 | Kalka_070 | **2.33^E-05^** |
| V_cmax_ | 27.5 | Oyali_107 | 35 | Kalka_070 | 0.064 |
| V_cmax_ | 35 | Oyali_107 | 35 | Kalka_070 | **0.043** |
| V_cmax_ | 27.5 | Oyali_107 | 20 | Oyali_107 | **0.031** |
| V_cmax_ | 35 | Oyali_107 | 20 | Oyali_107 | **<0.00001** |
| V_cmax_ | 35 | Oyali_107 | 27.5 | Oyali_107 | **3.39^E-05^** |
| J_max_ | 27.5 | Kalka_070 | 20 | Kalka_070 | 0.33 |
| J_max_ | 35 | Kalka_070 | 20 | Kalka_070 | **0.00028** |
| J_max_ | 20 | Oyali_107 | 20 | Kalka_070 | 0.57 |
| J_max_ | 27.5 | Oyali_107 | 20 | Kalka_070 | **0.00070** |
| J_max_ | 35 | Oyali_107 | 20 | Kalka_070 | **2.56^E-05^** |
| J_max_ | 35 | Kalka_070 | 27.5 | Kalka_070 | **0.040** |
| J_max_ | 20 | Oyali_107 | 27.5 | Kalka_070 | 0.99 |
| J_max_ | 27.5 | Oyali_107 | 27.5 | Kalka_070 | 0.087 |
| J_max_ | 35 | Oyali_107 | 27.5 | Kalka_070 | **0.0040** |
| J_max_ | 20 | Oyali_107 | 35 | Kalka_070 | **0.015** |
| J_max_ | 27.5 | Oyali_107 | 35 | Kalka_070 | 0.99 |
| J_max_ | 35 | Oyali_107 | 35 | Kalka_070 | 0.91 |
| J_max_ | 27.5 | Oyali_107 | 20 | Oyali_107 | **0.036** |
| J_max_ | 35 | Oyali_107 | 20 | Oyali_107 | **0.0014** |
| J_max_ | 35 | Oyali_107 | 27.5 | Oyali_107 | 0.75 |
| R_d_ | 27.5 | Kalka_070 | 20 | Kalka_070 | 0.97 |
| R_d_ | 35 | Kalka_070 | 20 | Kalka_070 | 0.99 |
| R_d_ | 20 | Oyali_107 | 20 | Kalka_070 | 0.99 |
| R_d_ | 27.5 | Oyali_107 | 20 | Kalka_070 | 0.99 |
| R_d_ | 35 | Oyali_107 | 20 | Kalka_070 | 0.98 |
| R_d_ | 35 | Kalka_070 | 27.5 | Kalka_070 | 0.99 |
| R_d_ | 20 | Oyali_107 | 27.5 | Kalka_070 | 0.98 |
| R_d_ | 27.5 | Oyali_107 | 27.5 | Kalka_070 | 0.99 |
| R_d_ | 35 | Oyali_107 | 27.5 | Kalka_070 | 0.69 |
| R_d_ | 20 | Oyali_107 | 35 | Kalka_070 | 0.99 |
| R_d_ | 27.5 | Oyali_107 | 35 | Kalka_070 | 0.99 |
| R_d_ | 35 | Oyali_107 | 35 | Kalka_070 | 0.89 |
| R_d_ | 27.5 | Oyali_107 | 20 | Oyali_107 | 0.99 |
| R_d_ | 35 | Oyali_107 | 20 | Oyali_107 | 0.96 |
| R_d_ | 35 | Oyali_107 | 27.5 | Oyali_107 | 0.79 |

**References**

1. Karger, D. N., Nobis, M. P., Normand, S., Graham, C. H. & Zimmermann, N. E. CHELSA-TraCE21k v1. 0. Downscaled transient temperature and precipitation data since the last glacial maximum. *Clim. Past Discuss.* 1–27 (2021).

2. Lembrechts, J. J. *et al.* SoilTemp: a global database of near‐surface temperature. *Glob. Chang. Biol.* (2020) doi:10.1111/gcb.15123.

3. Abatzoglou, J. T., Dobrowski, S. Z., Parks, S. A. & Hegewisch, K. C. TerraClimate, a high-resolution global dataset of monthly climate and climatic water balance from 1958-2015. *Scientific Data* **5**, 170191 (2018).
